# The role of microvesicles and its active molecules in regulating cellular biology

**DOI:** 10.1111/jcmm.14667

**Published:** 2019-09-27

**Authors:** YingMei Lv, Jin Tan, Yuyang Miao, Qiang Zhang

**Affiliations:** ^1^ Department of Geriatrics Tianjin Geriatrics Institute Tianjin Medical University General Hospital Tianjin China; ^2^ Tianjin Medical University Tianjin China

**Keywords:** active molecules, apoptosis, autophagy, inflammation, lipids, microvesicles, miRNA, proteins

## Abstract

Cell‐derived microvesicles are membrane vesicles produced by the outward budding of the plasma membrane and released by almost all types of cells. These have been considered as another mechanism of intercellular communication, because they carry active molecules, such as proteins, lipids and nucleic acids. Furthermore, these are present in circulating fluids, such as blood and urine, and are closely correlated to the progression of pathophysiological conditions in many diseases. Recent studies have revealed that microvesicles have a dual effect of damage and protection of receptor cells. However, the nature of the active molecules involved in this effect remains unclear. The present study mainly emphasized the mechanism of microvesicles and the active molecules mediating the different biological effects of receptor cells by affecting autophagy, apoptosis and inflammation pathways. The effective ways of blocking microvesicles and its active molecules in mediating cell damage when microvesicles exert harmful effects were also discussed.

## 1 INTRODUCTION

Microvesicles (MVs) are a kind of nanoscale membrane vesicles released during cell activation, apoptosis and mechanical injury, and these are collectively called extracellular vesicles with exosomes and apoptotic bodies. In as early as 1946, Chargaff et al^1^ first discovered that plasma contains a subcellular ‘factor’ that can promote thrombosis, and subsequent studies have mostly used extracellular vesicles to describe this kind of substance. In recent years, the study of exosomes has become more and more mature, while unknown MVs have attracted more and more attention. It was found that MVs carry proteins, lipids, nucleic acids and other active components expressed in source cells, which can promote coagulation, participate in immunomodulation, induce angiogenesis and initiate apoptosis after interactions with target cells.^2^ Furthermore, these play an important role in a variety of diseases (such as cardiovascular disease,^3^ tumour, kidney disease and immune disease).

Previous studies have shown that MVs exert adverse biological effects when it interacts with target cells. For example, in cardiovascular disease, MVs can cause myocardial hypertrophy and mediate the progression of atherosclerosis and heart disease.^4^ In ischaemic encephalopathy, MVs can promote the progression of ischaemic encephalopathy.^5^ MVs can induce target cell injury by reducing cell viability,^6^ promoting cell dysfunction and inflammation after interaction with cardiomyocytes,^7^ endothelial cells and nerve cells. The investigators considered that the damage of MVs may be correlated to the bad state of the source cells. In recent years, it has been found that MVs from mesenchymal stem cells can prevent unilateral ureteral obstruction^8^ and that endothelial progenitor cell (EPC)–derived MVs have played a protective role in renal ischaemia‐reperfusion injury.^9^ Furthermore, adipose tissue mesenchymal stem cell–derived MVs have effects of anti‐inflammatory and cartilage protection.^10^ The protective effect of MVs may be attributed to the fact that its parent cells are stem cells with regenerative and repairing effects. As a carrier of transmission between cells, MVs carry specific active components of stem cells, and targets and transfers these protective substances, which causes the biological effects of cells to change to a beneficial direction. Therefore, it was considered that the different functions of MVs may be correlated to its active components.

In general, MVs in different cells in body fluids play a specific role. This role is mainly correlated to the various active components carried by MVs. The present study reviews the mechanism of the biological effects of MVs and its related active molecules in vivo, and the effective ways to alleviate the adverse effects of MVs. The aim of the present study was to explore the mechanism of MVs in regulating cellular biological effects and provide a theoretical basis for finding new therapeutic schemes for clinical diseases.

## 2 MVS AND ITS ACTIVE MOLECULES

### 2.1 Characteristics of MVs under different conditions

Microvesicles are spherical membranous vesicles encapsulated by a lipid molecular layer, and the cell spontaneously or, under certain conditions, the cell membrane phosphate ester serine valgus, which is redistributed to the outer side of the membrane in the bud and is released to the cell outside the subcellular component.^11^ MVs have a diameter of approximately 0.1‐1.0 μm and contain large number of bioactive carriers (protein, lipids, nucleic acids, etc). Furthermore, MVs play an important role in body fluids and tissues. Studies have shown that MVs can be derived from many types of cells, such as endothelial cells, erythrocytes, leucocytes, platelets and nerve cells,^12^ and in response to different stimuli, the release level of MVs in diseases is significantly higher than normal levels, such as the elevated level of endothelial microvesicles (EMVs) in cardiovascular disease,^13^ and hepatocyte from patients with hepatocellular carcinoma releases more MVs than normal hepatocytes.^14^ These phenomena indicate that MV release is correlated to the risk factors of cell exposure. Different environmental stimuli can not only change the number of particles, but also change the active ingredients carried by particles. An interesting study revealed that the co‐culture of normal bone marrow mesenchymal cell–derived MVs with multiple myeloma cells can reduce the viability, proliferation and migration of multiple myeloma cells, while MVs from multiple myeloma patients can enhance these biological effects.^15^ This shows that MVs produced in diseases are not only different in number from the normal state, but also have different biological effects. The main reason for these different effects is correlated to the changes in bioactive components carried by MVs.

Microvesicles come from many types of cells, carry active ingredients that have similarity with maternal cell components and contain some active analogous components and specific markers of the source cells.^16^, ^17^ The data are summarized in Table 1. Therefore, the same cell‐derived MVs should carry the same active components of parent cells. However, an interesting phenomenon was found. It was detected that MVs derived from apoptosis mainly carried CD31/annexin V, while MVs produced when endothelial cells were activated mainly carried CD105 or CD62E.^18^ In addition, the components of endothelial MV expression in different diseases are not the same. For patients with sleep disorders, EMVs mainly carried CD3, CD62E, CD42B, PECAM and E‐selectin.^19^, ^20^ In acute ischaemic stroke, EMVs mainly carried CD105, CD54, CD45, CD144, CD41A and CD4.^21^ In viral myocarditis, EMVs mainly carry CD31, CD144, CD3 and CD62E.^22^ This phenomenon reveals that in same cells in different environments or disease conditions, the derivative of MV‐borne active molecules varies. Therefore, there is reason to consider that MVs carry different active molecules and play an important role in different biological effects in cells.

**Table 1:** Bioactive substances produced by MVs from different cell sources and their biological effects on target cells

Cell‐derived microvesicles	Active molecule	Biological effect	Reference
Leucocyte	CD62L, PF4, TF, CD45, CD66b, CD11b, CD62p, CD31	Participates in thrombosis, regulates inflammatory response and vascular function	16, 17, 18, 19
Endotheliocyte	VCAM‐1, CD31, CD105, CD309, MadCAM1, CD51, CD146, CD62E, CD142, TF, miRNA‐19b24, PSGL‐1, CD144	Participates in endothelial dysfunction, angiogenesis, tumour growth, oxidative stress and so on	17, 20, 21, 22, 23, 24, 25, 86
Platelet	CD41, CD42A, TF, CD62P, CD40L, miRNA‐24, miRNA‐142‐3p, CD41b, CD61, CD63, CD11b, RANTES, NF‐KB, GPIbα, GPIIb/IIIa	Participates in promoting coagulation, promoting thrombosis and promoting apoptosis	17, 18, 23, 27, 28, 29, 30, 32, 60, 83
Erythrocyte	CD44, CD47, CD55, TF, PS, CD235a	Mediated coagulation reaction	30, 31, 83
Neutrophils	Ly6G, CD41, Ter119, CD31, CD142, CD45, CD66b, IL‐1β	Mediates inflammation and subsequent vascular damage	17, 84, 87

Abbreviations: GPIbα, GPIIb/IIIa, platelet‐specific autoantibody; MadCAM1, mucosal addressin cell adhesion molecule‐1; PF4, platelet factor 4; RANTES, regulated upon the activation of normal T cell expressed and secreted; VCAM‐1, vascular cell adhesion molecule‐1.

### 2.2 Active molecules carried by MVs

Microvesicles comprise of a phospholipid bilayer derived from the parental cell plasma membrane, secretory proteins and genetic material transmitted in the cytoplasm. These specific molecules derived from parental cells are packaged in a bilayer structure or carried on the membrane surface, which is called active ingredients. In recent years, MVs related to active molecules have been classified into three main categories: protein, lipid and nucleic acid. Lipids are derived from parental cell membrane components, which mainly constitute the MV skeleton and extracellular nucleotide signals. Proteins mainly come from parental cell membranes and the cytoplasm. It has been revealed that cytotoxic proteins of natural killer cells are mainly correlated to the cytotoxic proteins contained in these. Microvesicles derived from natural killer cells act as a carrier for long‐distance communication. The mass spectrometry analysis revealed that it contains cytotoxic cell–specific protein granzyme A and other protein components (such as heat‐shock protein, ubiquitin‐proteasome system component, protein biosynthetic enzyme, energy metabolism enzyme, nuclear protein, serum protein and cytoskeleton protein).^23^ This indicates that the active protein carried by MVs is consistent with the components expressed by parental cells. In addition, MVs have also been considered to the main carrier of functional mRNA horizontal metastasis between cells, including large number of parental cell mRNAs.^24^ The main reason is that MVs engulfs some cytoplasm during production, which contains the protein and mRNA from parental cells. Various types of active molecules expressed in MVs come from different parts of cells, which involve various regions, such as the plasma membrane, cytoplasm and nucleus. These are important factors that affect intercellular signal transduction. The data are summarized in Figure 3.

As confirmed by multiple pathological conditions, the active substances carried by MVs participate in various biological effects in cells. For EPCs, MVs derived from EPCs can increase the proliferation rate of renal tubular cells and decrease the apoptosis and reactive oxygen species (ROS) of human brain microvascular endothelial cells under hypoxia‐reoxygenation. Hence, the protective effects of MVs treated with RNA enzymes are significantly weakened. These protective effects of MVs were significantly weakened after treatment with RNA enzymes.^9^, ^25^ The decrease in the biological effect of MVs suggests that the RNA, which is the active component of MV metastasis, is the key factor of MVs and plays a protective role. When the active molecules were inhibited, the effect of MVs on the biological effects of receptor cells was significantly reduced.

## 3 BIOLOGICAL EFFECTS OF ACTIVE MOLECULES

### 3.1 Lipid active molecules and their biological effects

**Figure 1:**
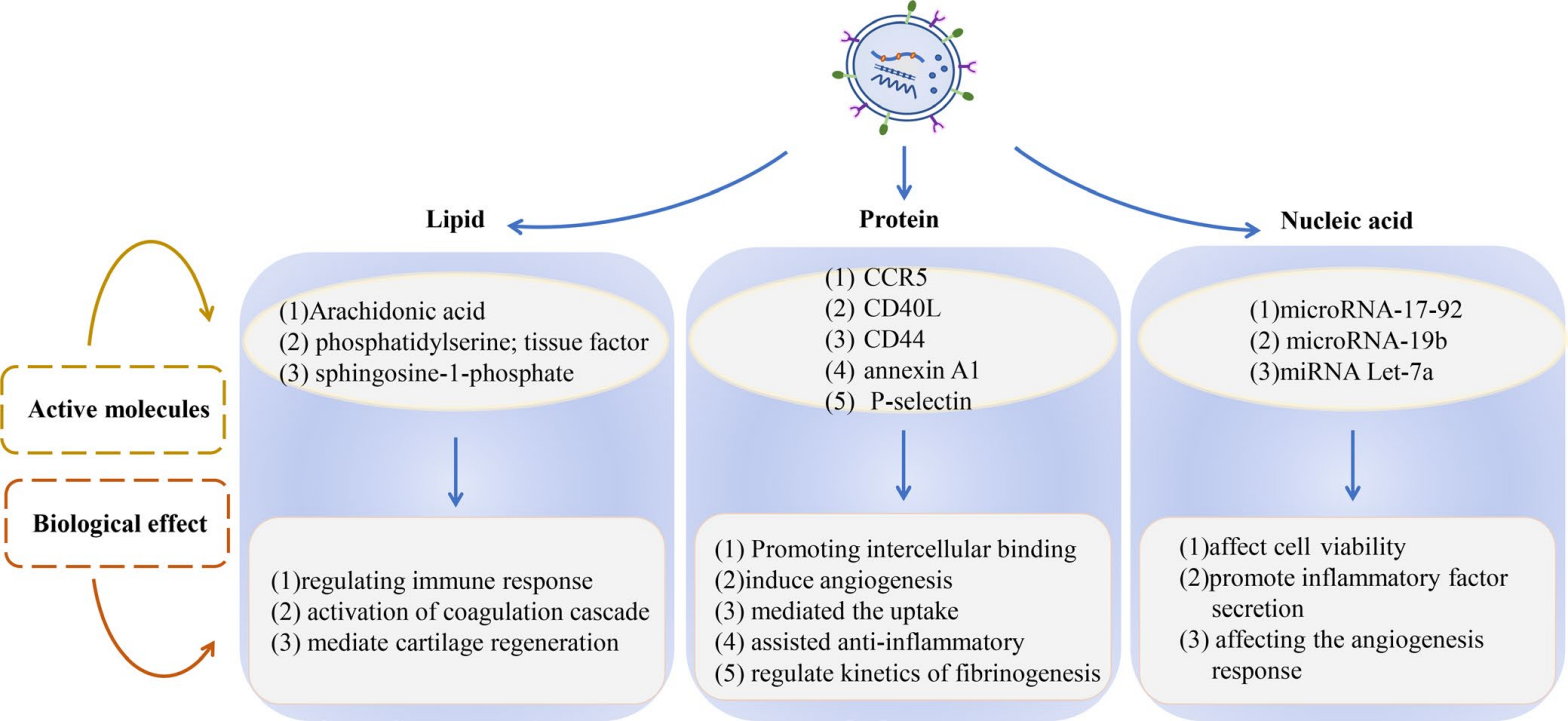
Cell‐derived MVs mainly carry three types of active substances: protein, lipid and nucleic acid. Among these, the different effects of MVs in target cells are mainly due to the different active ingredients these carry. As shown in this figure, the effects of MVs, which carry different types of active substances in target cells, are listed

Lipids expressed by MVs mainly come from the plasma membrane components of parental cells, including sphingosine‐1‐phosphate, phosphatidylserine (PS), cholesterol and arachidonic acid. These active lipids have many biological functions in body, such as asthma, cancer, haemostasis, immunity and inflammation. The data are summarized in Figure 1. Arachidonic acid and other polyunsaturated fatty acid signalling molecules have been identified as biomarkers and determined to be involved in regulating distal immune response.^26^, ^27^ In addition, the pro‐coagulant cascade and mediating cartilage regeneration are also important physiological functions of active lipids. Phosphatidylserine and tissue factor (TF), which are expressed by MVs, are directly involved in the activation of the coagulation cascade in the body.^28^ TF is inhibited in resting cells. Furthermore, sphingosine is hydrolysed after stress injury, thereby eliminating the inhibition of TF, which leads to TF activation,^29^ and affecting the pro‐coagulant effect of receptor cells. Microvesicles that contain arachidonic acid released after platelet activation also have this pro‐coagulant effect after being ingested by endothelial cells.^30^, ^31^ High levels of sphingosine‐1‐phosphate in bone marrow mesenchymal stem cell (MSC)–derived MVs can mediate cartilage regeneration.^32^ Furthermore, various active molecules can be transferred to receptor cells with the uptake of MVs by cells, mediating changes in cellular biological effects. The biological effects of MVs with different active lipids are different in vivo.

### 3.2 Protein active molecules and their biological effects

Cells release MVs that carry active molecules from parental cells under various stimulations, including content proteins and surface proteins. The protein active components expressed by MVs can interact with target cells, promote the adhesion and internalization of MVs to target cells, induce angiogenesis and regulate coagulation reactions, such as CD44 and P‐selectin. Microvesicles carrying chemokine CCR5 can interact with monocytes or T cells, which lack CCR5 and induce the biological effect of CCR5 in cells, and promote the binding of macrophages to HIV‐1 virus.^33^ Some specific protein components on the surface of MVs, such as ligand CD40 (CD40L), can stimulate endothelial cells to induce angiogenesis.^34^ MVs that express CD44 mediate the uptake by human chondrocytes,^32^ while up‐regulated annexin A1 in MVs assists in anti‐inflammatory and cartilage protection.^10^ In addition, The P‐selectin contained in platelet‐derived microvesicles (PMVs) could improve the kinetics of fibrinogenesis and normalize the bleeding time of haemophilia mice by targeting MVs to the injured site. It is mainly due to the combination of P‐selectin and P‐selectin glycoprotein ligand 1 (PSGL‐1) that allows for the recruitment of MVs to express TF and subsequently affects the blood coagulation state.^35^ When the internal environment changes, the biological effects of P‐selectin on coagulation would also change MVs in carrying proteins, including signal molecules, receptors, integrins and cytokines, which play an important role in regulating and assisting the progression of cell life in all aspects, and are the main dependent components of normal cell activities.

### 3.3 Nucleic acid active molecules and their biological effects

Nucleic acid is one of the most basic substances to maintain normal life. It is also one of the most important active components carried by MVs, which include microRNA, mRNA, RNA and DNA. As a genetic material of parental cells, nucleic acid active molecules are mainly responsible for the transmission of genetic signals between cells and the regulation of protein synthesis. Microvesicles transfer nucleic acid to the target cells through receptor ligand binding, membrane fusion and endocytosis. When the genetic material enters the target cells, it regulates the gene expression and protein synthesis of target cells and affects the biological effects. Microvesicles carry and transfer genetic material to target cells, which has been confirmed in many studies. Among these, microRNA‐17‐92 clusters carried by MVs were considered to affect the viability of target cells in a dose‐dependent manner, after entering the target cells.^36^ EMVs carrying microRNA‐19b into perivascular adipose tissue were considered to promote the secretion of inflammatory cytokines and macrophage infiltration in adipose tissues.^37^ Transferring microRNAs from platelet MVs to tumour cells and endothelial cells has been considered to induce apoptosis.^38^, ^39^ Studies have revealed that MVs affect gene expression in target cells by transferring nucleic acid components. There are two main methods: (a) When the nucleic acid components carried by MVs enter the target cells, these affect the gene programming of target cells and regulate protein translation by regulating the microRNAs of receptor cells. (b) MVs transfer microRNAs directly into target cells and translates these into proteins, affecting gene expression biological functions. Platelet‐derived microvesicles were incubated with human umbilical vein endothelial cells (HUVECs), internalized by HUVECs, and the carrying microRNA let‐7a was transferred to HUVECs. The active substance microRNA let‐7a can directly target the 3′ untranslated regions (3′UTRs) of THBS‐1 and inhibit the release of THBS‐1, affecting the angiogenesis response.^40^ In addition, PMV infiltration in solid tumours, the anchoring to tumour cells and the metastasis of microRNAs result in the gene suppression of tumour cells, which can inhibit the growth of primary tumours and affect the immune response, the angiogenesis of tumours and other cancer‐related processes. At the same time, other active ingredients (including growth factors, angiogenesis regulators, second messengers and lipids) carried by MVs are also transported to receptor cells along with the transfer of nucleic acid, which promotes the change in biological effects of receptor cells.^41^

## 4 MECHANISM OF MVS AND ITS ACTIVE MOLECULES IN EXERTING BIOLOGICAL EFFECTS

**Figure 2:**
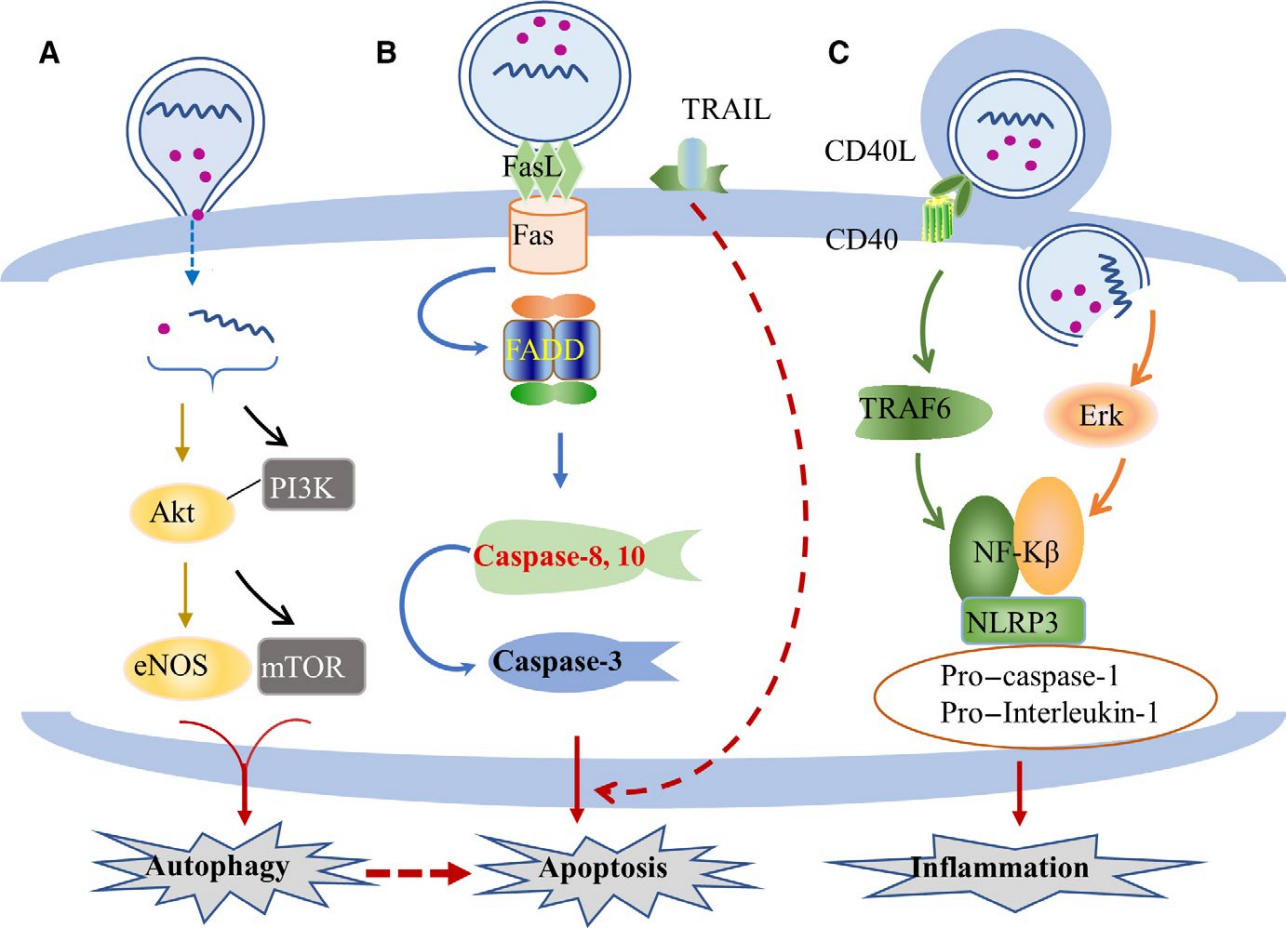
The three main pathways for the biological effects of target cells mediated by MVs and their active substances: (A) regulate autophagy through the PI3K/Akt pathway; (B) regulate the apoptosis through the Fas/FasL pathway; and (C) regulate the inflammatory through the NF‐κB pathway

Under physiological and pathological conditions, MVs are involved in the changes in biological effects of many kinds of cells, although the mechanism of MVs and its active molecules remain unclear. However, it has been proven that MVs can participate in the pathophysiological process of different organs by transferring active molecules, such as microRNA, proteins and lipids. The transfer of active molecules to receptor cells after MVs interact with cells has been confirmed by many researchers. Mesenchymal cells regulate intercellular immune response by transferring MVs and its carrying microRNAs.^42^ Similarly, MVs isolated from human atherosclerotic plaques transfer intercellular cell adhesion molecule‐1 (ICAM‐1) to endothelial cells,^43^ increase the affinity of endothelial cells, facilitate monocyte adhesion and promote the progression of atherosclerotic plaques. For MVs, the transferring of active substances into target cells can activate the signal transduction, affecting the biological function of receptor cells. At present, there are three main pathways of MV activation: (a) PI3K/Akt‐mediated autophagy, (b) Fas/FasL‐mediated apoptosis and (c) NF‐KB–mediated inflammation. The data are summarized in Figure 2.

### 4.1 MVs and its active molecules regulate autophagy through the PI3K‐AKT pathway

When MVs and its active molecules are absorbed by target cells, these can activate a signal cascade reaction in target cells and regulate the biological response. The PI3K‐Akt pathway is the most important signalling pathway in mammalian autophagy. It is involved in the regulation of cell proliferation, differentiation, apoptosis and glucose transport by influencing the activation of effector molecules downstream. Microvesicles transmit maternal active molecules and affect the biological effects of target cells by activating the PI3K‐Akt signalling pathway. In cancer cells, MVs carry hyaluronic acid. After the uptake by monocytes, the expression of PI3K and Akt increases, while the downstream factor mTOR is inhibited, indicating the activation of autophagic signalling pathway. It was further revealed that the synthesis of IL‐10 in monocytes also significantly increased. As a negative regulator, IL‐10 plays an important role in regulating the inflammatory response of cancer cells, indicating that MV transmission is involved in the biological effects of cancer cells by inducing the activation of autophagic pathway changes.^44^ In EPCs, MVs promote angiogenesis and maintain β cell function in islet endothelial cells by activating the PI3K signal. The activation of the PI3K signal may be due to the MVs that carry microRNAs (microRNAs‐126 and microRNAs‐96) from parental cells.^45^ After entering the target cells, exogenous microRNAs affect gene expression and PI3K‐Akt signal transduction. Furthermore, the activated PI3K‐Akt signal acts on the downstream substrate endothelial nitric oxide synthase (eNOS). The eNOS is an important regulator of angiogenesis and vascular tension, which can trigger the process of angiogenesis, leading to cell proliferation, differentiation and angiogenesis.^46^ MVs activate the PI3K‐Akt autophagy signalling pathway, and play a protective role in target cells.^47^ However, when the autophagy signal is overexpressed or autophagy is inhibited,^48^, ^49^ MVs can induce apoptosis and damage cells. Different cell‐derived MVs and its expressed active molecules activate the PI3K‐Akt pathway in receptor cells and regulate target cells to produce different biological effects.

### 4.2 MVs and its active molecules regulate apoptosis through the Fas/FasL pathway

FasL is a cytokine that can bind to the death receptor TNFRSF6/FAS. In recent years, Fas and its ligand FasL been the most deeply studied membrane surface molecules related to apoptosis. FasL and TNF‐related apoptotic‐inducing ligand (TRAIL) are two of the known mediators that induce cell death.^50^, ^51^ FasL recognizes Fas on the surface of target cells and triggers apoptotic processes, such as the activation of caspase‐3, which subsequently induces programmed cell death.^52^ This is mainly because FasL binds to Fas, binds to the death domain region of the adaptor protein FADD and binds subsequently to the N‐terminal D of FADD. The death effector domain binds to the caspase‐8 (or caspase‐10) precursor protein, which activates caspase‐8 and caspase‐10 through self‐shearing. These initiate the caspase cascade reactions, activate caspase‐3, degrade intracellular structural and functional proteins, and ultimately lead to apoptosis.^53^, ^54^, ^55^ At the same time, the TRAIL carried by MVs may participate in and cooperate with the apoptotic effect of FasL.^56^ FasL not only induces apoptosis, but also promotes inflammation.^57^ Hence, this may be involved in another new pathway of programmed cell death induced by intracellular inflammation (Figure 2).

Circulating MVs transfer caspase‐3 to target cells, regulate TNF‐α and TRAIL signalling pathways, cause cell death and promote the development of cardiovascular diseases.^58^ Furthermore, MVs carrying apoptosis‐related spot‐like protein (ASC) also have an apoptotic effect. ASC plays an important role in activating caspase‐1, which is different from FAS/FASL‐induced programmed cell death and considered to be the pathological death of cells under inflammatory and stress conditions.^59^

### 4.3 MVs and its active molecules regulate inflammation through the NF‐κB pathway

Nuclear factor‐kappa B (NF‐κB) exists in almost all animal cells and mainly plays the role of transcription factor. It can regulate gene expression and affect various biological processes, including innate and adaptive immunity, inflammation, stress response, B cell development and lymphoid organ formation. Microvesicles carry active molecules from parental cells into target cells by mediating the activation of NF‐κB, resulting in changes in biological effects. For example, PMVs carry GPIbα, GPIIb/IIa, PS, P‐selectin and CD40L from maternal cells. These surface molecules confer PMV pro‐inflammatory and pro‐coagulant properties. Hence, how can MVs achieve this pro‐inflammatory effect? It is self‐evident that the activation of NF‐κB plays an important role in this process. It was found that CD40L transferred by MVs could bind to CD40 on the surface of monocyte membranes and trigger the activation of downstream signal molecule TNF receptor–associated factor 6 (TRAF6). As a downstream substrate of TRAF6, NF‐κB could be simultaneously activated. This also promotes the release of inflammatory mediators from monocytes.^60^ In addition, PMVs and its active substances can also induce the activation of the NF‐κB factor by activating Erk, the upstream active molecule of NF‐κB, and mediate the adhesion, movement and inflammation of rheumatoid arthritis fibroblast‐like synoviocytes (RA‐FLS). A variety of inflammatory cytokines and chemokines secreted by RA‐FLS have been considered as the key factors to promote the progression of rheumatoid arthritis.^61^, ^62^ NF‐κB can mediate the inflammatory response of various cells mainly because the activation of NF‐κB mediates the formation of inflammatory corpuscle complexes of NLRP3, pro–caspase‐1 and pro–interleukin‐1, which in turn promotes the release of IL‐1β. In the regulation of biological effects of receptor cells, MVs, as an important carrier of intercellular communication, have far‐reaching effects on peripheral cells and distant target cells.

In addition, MVs not only mediate inflammation through the NF‐κB signalling pathway, but also activate similar pro‐inflammatory responses in target cells through a variety of pathways. For example, MVs enhance lung inflammation by carrying IL‐1β and TNF‐α,^63^ or increase the expression level of mRNA in receptor cells. Circulating MVs bind to the cell membrane and transfer monomer C‐reactive proteins to the cell surface. This converts pentameric C‐reactive protein into pro‐inflammatory monomer C‐reactive proteins after myocardial infarction, and promotes inflammation.^64^ The study conducted by De Souza et al^65^, ^66^ revealed that MVs can regulate the activation of NF‐κB when it transported microRNAs (microRNA‐27) expressed in parental cells into the target cell. Although its specific mechanism remains unclear, the role of MVs in it is self‐evident. Various cytokines and pathways regulate inflammatory pathways in cells. These are closely correlated to signal transduction pathways, such as autophagy and apoptosis, and together maintain effective changes in cellular biological effects.

## 5 EFFECTIVE WAYS OF BLOCKING CELL DAMAGE MEDIATED BY MVS AND ITS ACTIVE MOLECULES

**Figure 3:**
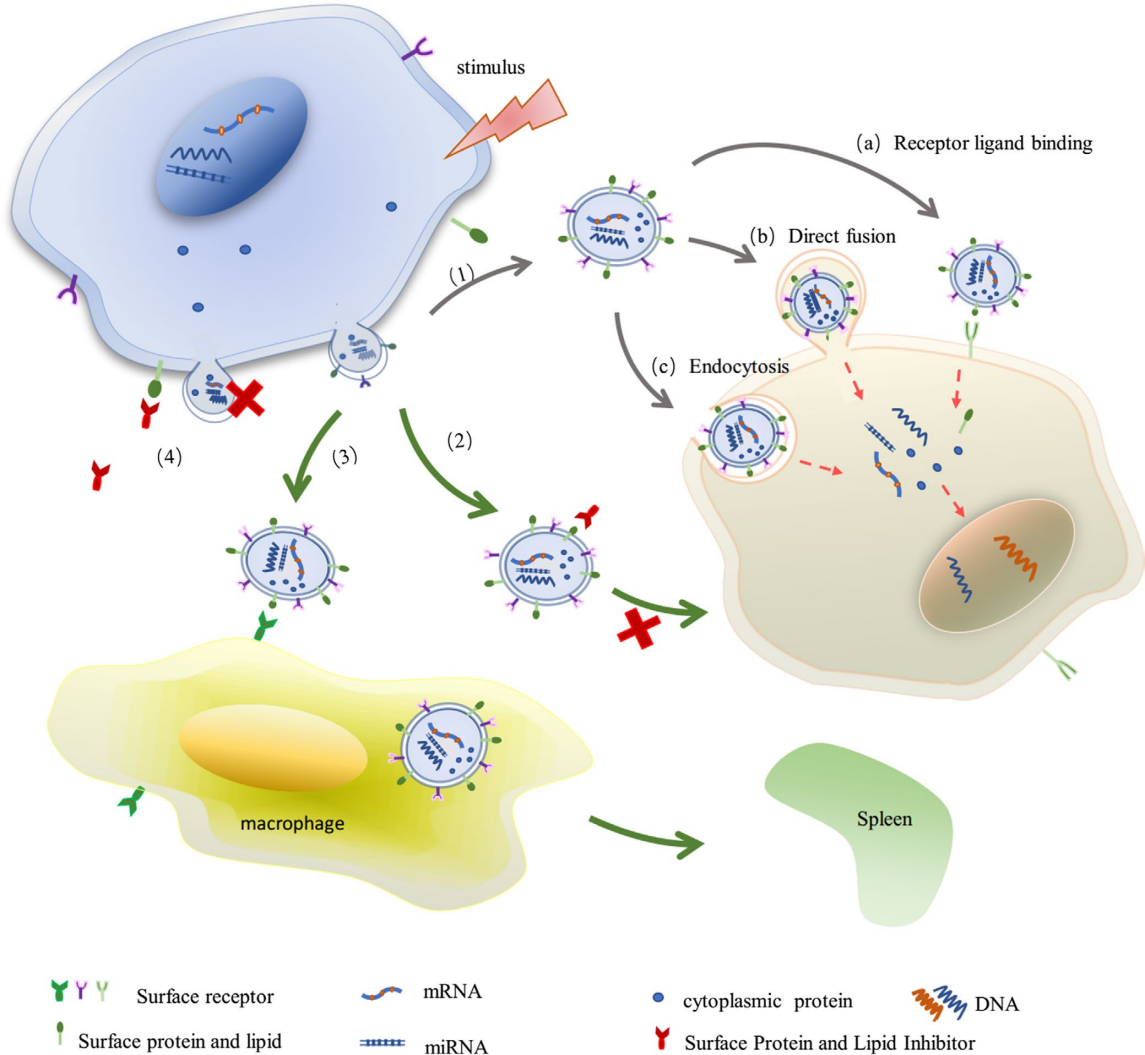
Under stress, cells release spherical MVs containing parental cell‐active components, in which the lipid carried by MVs originates from the plasma membrane, while the protein mainly originates from the cell surface membrane and cytoplasmic secretory proteins, and nucleic acid from the nucleus. (1) These active substances are transferred to target cells through (a) receptor ligand binding, (b) direct fusion and (c) endocytosis, regulating gene expression and protein synthesis in target cells. Effective ways to reduce the content of MVs in body fluid are as follows: (2) inhibiting the interaction between MVs and target cells, and downstream signal transduction; (3) mediating MV clearance; and (4) inhibiting the release of MVs

As an important mediator of intercellular signal transduction in body fluids, MVs play an important role in the occurrence and development of diseases by regulating autophagy, apoptosis and other signalling pathways, and participating in cell proliferation, differentiation, death and other biological reactions. In view of this biological effect of MVs, it can be determined that the target of blocking the adverse effect of MVs in clinic is by mastering its biological mechanism and important sites, providing a new direction for the treatment of diseases. There are three effective ways to block MVs and its active substances that mediate cell injury: (a) blocking the expression of active molecules carried by MVs and signal transduction in target cells; (b) mediating MV clearance; and (c) reducing the release of harmful MVs. The data are summarized in Figure 3.

### 5.1 Blocking the interaction of MVs and its active molecules with receptor cells and transduction of the downstream signalling pathway

Microvesicles mediate the biological changes in target cells through a variety of ways. Hence, there are many methods to block the interaction between MVs and its active substances and cells, including blocking the expression of active substances or inhibiting the activation of key factors in MVs mediated by the signalling pathway. This can alleviate the adverse effects of MVs on cells. For example, when drugs were given to inhibit cholesterol synthesis, the expression of cholesterol in MVs decreased, which reduced the binding and interaction between MVs and cells. When the expression of PS in MVs was blocked, the coagulation reaction in patients with non–ST‐segment elevation myocardial infarction after stent placement was alleviated.^67^ The reason is that the level of pro‐coagulation decreased with the decrease in PS expression. In addition, when MVs and its active substances mediated the activation of the target cell signalling pathway, blocking the transmission of the signalling pathway can alleviate the biological effects caused by MVs. Aspirin partially blocks the inflammatory, oxidative stress, coagulation and adhesion properties of MVs by inhibiting ErK‐NO/O_2_ and p38‐NF‐κB‐VCAM‐1 signalling pathways.^68^ Losartan, an angiotensin type 1 receptor (AT1 receptor), can inhibit MV‐induced endothelial cell senescence by reducing the expression of mitogen‐activated protein kinase and PI3K.^69^ MVs collected after the bortezomib and lenalidomide treatment of tumour cells inhibited the activation of NF‐κB in HUVECs and decreased the secretion of angiogenic factors.^70^ This provides another mechanism for antiangiogenesis therapy. Regardless of whether blocking the expression of active molecules or inhibiting the activation of key factors in the signalling pathway is mediated by MVs, they all play a therapeutic role in the progression of diseases. In clinic, blocking the transmission of bad signals between cells mediated by MVs has a certain therapeutic effect. However, as a new treatment to alleviate the progression of diseases, this still needs further exploration and research.

### 5.2 Mediated MV clearance

In response to various stimuli (such as oxidative stress, inflammation, vascular shear stress), the level of circulating MVs increases, causing a series of pathophysiological changes in the body. In order to maintain the stability of the internal environment, the body can remove these MVs to a certain extent, thereby reducing the adverse effects of MVs on cells and organs. Microvesicles from different cell sources in circulation can bind to cells that mediate its clearance and remove through the liver, spleen and lungs.^71^, ^72^ The clearance of MVs is associated with its surfactants and receptors expressed on the cell surface. Phosphatidylserine, which is carried on the surface of MVs, can bind to the developmental endothelial cell locus 1 (Del‐1) expressed on endothelial cells. This promotes the endothelial cell uptake and clearance of circulating MVs.^73^ HUVECs mediate the endocytosis and clearance of PMVs in a Del‐1–dependent manner. Phosphatidylserine on the surface of MVs binds to the lactadherin‐2C domain secreted by macrophages, and fixes MVs to macrophage integrin by RGD sequence. This promotes the removal of circulating MVs through macrophages, especially PMVs.^74^ In addition, the clearance of PMVs has been considered to be correlated to β‐2‐glycoprotein, which can mediate the phagocytosis of PMVs through THP‐1–derived macrophages and reduce the content of PMVs in circulation.^75^ Endothelial cells, macrophages and other phagocytes can clear up the increase in MVs in circulation and maintain a certain dynamic balance of circulating MV levels. This is also one of the ways to maintain homeostasis.

When circulating MVs are reduced to the normal level, some physiological changes in the body can be alleviated and improved to some extent, such as inflammation injury, and blood hypercoagulability. MerTK‐mediated alveolar macrophage phagocytosis can clear MVs in the lungs, which could alleviate pulmonary inflammatory damage.^76^ Del‐1 mediates the clearance of PMVs through endothelial cells, which can reduce the cytokine response of synovial fibroblasts and stimulate haematopoietic cells.^73^ Lactadherin mediates the macrophage clearance of PMVs, which reduces the hypercoagulability state of circulating blood.^74^ The consumption of EMVs in patients with acute coronary syndrome decreases the induction of ageing. Mediating the clearance of MVs can prevent the damage of its active substance to receptor cells and, at the same time, alleviate the adverse reactions of tissues and organs.

### 5.3 Reduce harmful MV release

In order to maintain the stability of the internal environment, the body clears the increased MVs in body fluids to a certain extent, but its compensatory ability is limited. When the level of MVs increases, the body cannot maintain the level of MVs in the normal range for a long time. At this time, reducing the release of MVs has been considered to be one of the effective ways to reduce MV content in circulation. In clinic, some therapeutic drugs, such as aspirin, imipramine, acetylsalicylate and resveratrol, have exhibited obvious effects in reducing the release of MVs in circulation, and their ability to alleviate the progression of diseases to a certain extent. For example, after aspirin treatment, PMVs decreased, and the expression of ERK1/2, p38 MAPKs and JNKs decreased, thereby reducing the damage of MVs to target cells.^68^ Aspirin also inhibits the activation of vascular parietal cells by reducing the release of MVs.^77^ Resveratrol can reduce the levels of TNF‐α and EMVs in peripheral blood by a cascade of intracellular NF‐κB molecules and has a good effect on vascular endothelial function and systemic inflammation.^68^ Acetylsalicylate treatment can reduce the number of circulating endothelial cells and PMVs in patients, and has been widely used in the treatment of cardiovascular diseases.^78^ Studies have shown that the dietary intake of polyunsaturated fatty acids, fruits and vegetables can reduce leucocyte activation, platelet aggregation and MV shedding, thereby reducing the risk of cardiovascular disease and delaying the progression of atherosclerotic thrombosis.^79^, ^80^ Some scholars have revealed that after providing calcium chelating agent EGTA, the biosynthesis of MVs was significantly reduced.^81^ Deferiprone can inhibit iron‐induced ROS production, reduce endothelial cell apoptosis and EMV release, and protect endothelial cells from iron‐induced toxicity.^82^ This reduces the content of various MVs in circulation and alleviates the damage of MVs to cells. In conclusion, drugs and reasonable diet can block the release of MVs and reduce the content of MVs in circulation, thereby alleviating the damage of MVs to cells.

In addition, most scholars have considered that the protective effect of stem cell MVs on mitigating receptor cell injury in vivo is probably due to the ability of MVs to carry parental microRNAs with regenerative function.^83^ In recent years, some scholars have applied MVs secreted by stem cells to investigate tissue cell damage in clinical treatment, and this has become an effective method to alleviate the brain damage caused by cancer radiotherapy.^84^ Stem cell MVs treatment is one of the effective ways to alleviate the progress of disease, and the therapeutic effect of stem cell MVs has been given more and more attention.

## 6 PROSPECTS

Cell‐derived MVs have received increasing attention in the scientific community due to their potential to serve as biomarkers of intracellular events and their innate biological activities. These extracellular vesicles have been initially considered as simple by‐products of pathological disorders. Subsequently, it was considered as another new substance that mediates signal transduction in vivo. Furthermore, it plays an important role in the pathogenesis and progression of atherosclerosis, stroke, coronary heart disease and diabetes mellitus by carrying proteins, lipids and nucleic acids expressed by maternal cells. As mentioned above, MVs mediate different biological responses by activating the PI3K‐Akt, Fas/FasL, and NF‐κB signalling pathways in receptor cells. However, more recent evidence has also shown that MVs are not necessarily harmful and are necessary to alleviate the state of the disease. This effect is closely correlated to the active components of parental cells carried by MVs. More and more studies have revealed that the molecular characteristics and cell origin of MVs reflect the nature of the disease itself, and are affected by the progression and treatment. This provides a powerful tool for diagnosis, prognosis and drug monitoring. However, our understanding of the contribution of MVs in the pathophysiological processes of human diseases and its potential mechanisms remains limited. Hence, new diagnoses or treatments that involve MVs are needed.

Various physiological factors and different pathological stimuli may affect the production and composition of MVs, resulting in different active substances carried by MVs and different biological effects. Therefore, the mechanism of MVs and its active substances in the body is the key to identify disease markers. Previous studies have revealed the abnormal levels of MVs expressing CD62E and MIL‐126‐3P in pre‐diabetic patients,^85^ which may be a marker of endothelial dysfunction. On the other hand, mastering the biological process of MVs and its active substances that act on target cells is the key to preventing and treating diseases. This is a new hope for clinical treatment, which may be applied to the treatment of diseases in the future.

## CONFLICT OF INTEREST

The authors declare no conflict of interest.

## AUTHOR CONTRIBUTIONS

Conception and design: YingMei Lv, Jin Tan, Qiang Zhang. Wrote the paper: YingMei Lv. Language modification and guidance: Yuyang Miao. All authors have read and approved the final manuscript.

## Data Availability

The data that support the findings of this study are available from the corresponding author upon reasonable request.
